# First-trimester diagnosis and management of Cesarean scar pregnancies after in vitro fertilization-embryo transfer: a retrospective clinical analysis of 12 cases

**DOI:** 10.1186/s12958-015-0120-2

**Published:** 2015-11-21

**Authors:** Yan Ouyang, Xihong Li, Yan Yi, Fei Gong, Ge Lin, Guangxiu Lu

**Affiliations:** Institute of Reproductive and stem cell Engineering, Central South University, Xiangya Road, Changsha, Hunan 410008 P.R. China; Reproductive and Genetic Hospital of Citic-Xiangya, Xiangya Road, Changsha, Hunan 410078 P.R. China

**Keywords:** Cesarean scar pregnancy, Heterotopic cesarean scar pregnancy, First-trimester, Transvaginal sonography, Diagnosis, Management, In vitro fertilization-embryo transfer

## Abstract

**Background:**

Although Caesarean scar pregnancy (CSP) is rare, it can cause life-threatening complications. The increasing rate of Cesarean delivery plus rapid development of in vitro fertilization-embryo transfer (IVF-ET) may increase the occurrence of CSP as well as the ratio of heterotopic CSP (HCSP)/CSP. Therefore, early diagnosis and management of CSP are necessary to avoid serious complications. And the purpose of this article is to evaluate the importance and feasibility of the first-trimester diagnosis and management of CSP after IVF-ET.

**Methods:**

All the 12 cases were secondary infertility patients who had a history of Cesarean section and underwent IVF-ET in our reproductive center. All cases with CSP were diagnosed using transvaginal color Doppler sonography (TVS). Medical, surgical and expectant managements were implemented, and the management results were traced.

**Results:**

Patients with CSP (*n* = 12) were diagnosed from January 2011 to April 2015, 6 (50 %) of which were HCSP. The prevalence of CSP was 1:1688 pregnancies. The gestational age ranged from 5 + 3 to 7 + 4 weeks in all CSP, and from 5 + 6 to 7 + 4 weeks in HCSP at diagnosis. Five patients received successful surgical treatment. The success rate of medical and expectant management was 50 % (1/2) and 100 % (5/5), respectively. One patient with failed medical management needed an emergency laparotomy to evacuate CSP. The uterus was preserved in all 12 patients.

**Conclusions:**

The Caesarean section and IVF-ET may increase the ratio of HCSP/CSP. TVS is a noninvasive and effective tool for use in diagnosing CSP. CSP should be carefully excluded in patients who have had a history of Caesarean section. Early diagnosis of CSP in the first trimester may contribute towards the preservation of uterus as well as intrauterine pregnancy (IUP) in HCSP.

## Background

Caesarean scar pregnancy (CSP) is defined as an ectopic pregnancy (EP) implanted in the myometrium of a previous Cesarean section scar [1]. Ever since the first case of CSP was reported by Larsen and Solomon in 1978 [2], there has been increasing attention paid to this rare condition. Although many hypotheses have been proposed to explain the mechanism of CSP, its etiology is still unclear. One probable theory is the conceptus might migrate through a microscopic dehiscent tract from the endometrial canal to the scar tissue [3, 4]. The predisposing risk factors include uterine trauma, Caesarean section, myomectomy, in-vitro fertilization-embryo transfer (IVF-ET), manual removal of placenta, adenomyosis, etc. [5]. IVF-ET could be a sole risk factor even without any previous uterine surgery [6].

The incidence of CSP is extremely low, and has been estimated from 1:2216 to 1:1800 [3, 7, 8]. Although this entity is extremely rare, it can cause life-threatening complications such as uterine rupture and catastrophic hemorrhage which may relate to maternal and fetal morbidity and mortality, even at the early stage of gestation [1]. The rate of Cesarean delivery increases continuously over the years [3, 9]. Particularly in China, almost 50 % infants are delivered by Cesarean section, which may increase the occurrence of CSP [10, 11]. In addition, with the widespread use of IVF, the incidence of HP has increased to 1 %, which is estimated from 1:50,000 to 1:10,000 spontaneously [12, 13]. Therefore, the occurrence of heterotopic CSP (HCSP), one of the rarest forms of EP, is also increasing [14]. All of the above necessitate the early diagnosis and treatment of this special kind of EP to avoid serious complications and preserve patients’ fertility.

However, there has been no study focusing on the first-trimester diagnosis and management of CSP in IVF patients currently, and due to its rarity, no universal treatment guideline has been established until now. Herein, we evaluated 12 cases of pregnancies implanted into the previous Cesarean section scar after IVF-ET procedure in our unit over a 4-year period. All patients were detected by transvaginal sonography (TVS) and managed in the first trimester. We present our experience and evaluate the importance and feasibility of the first-trimester diagnosis and management of CSP after IVF-ET.

## Methods

From January 2011 to April 2015, 12 cases of CSP were diagnosed in the Reproductive and Genetic hospital of Citic-Xiangya. All 12 cases were secondary infertility patients who had a history of Cesarean section and had undergone IVF-ET at our reproductive center. The clinical data of the 12 patients were analyzed retrospectively in this study.

A routine blood test for serum beta human chorionic globulin (β-hCG) was performed on Day 14 after IVF-ET, and a routine TVS scan was scheduled on Day 28 post IVF-ET to examine the viability and location of pregnancies. GE VOLUSON E8/730 (GE Tech Co., Ltd., New York, America) equipped with a 5–9 MHz vaginal color Doppler probe was used. If patients complained of abdominal pain and/or vaginal bleeding, TVS examination would be arranged soon.

**Fig. 1:**
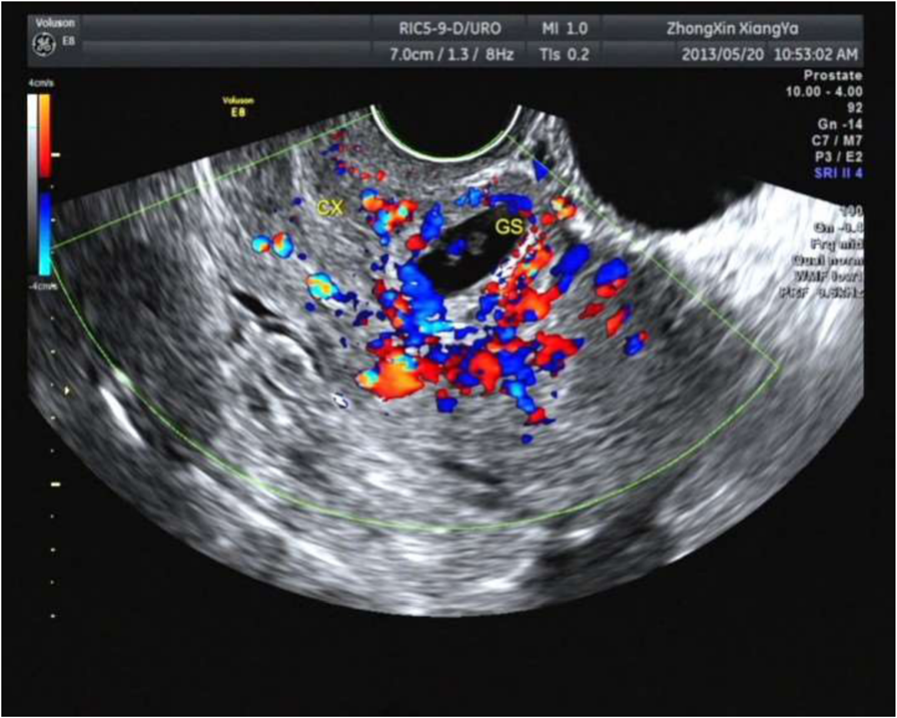
Ultrasound findings of a typical CSP. Longitudinal section of the uterus showing a 6 + 3 weeks with cardiac activity gestational sac (Case 1; crown–rump length:3.6 mm) implanted into a previous Cesarean section scar and protruding towards the urinary bladder with strong peripheral color doppler signals

**Fig. 2:**
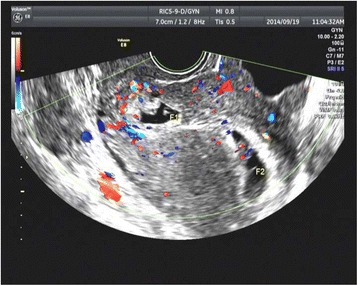
Ultrasound findings of a HCSP. Longitudinal section of the uterus showing the coexistance of an intrauterine pregnancy with a live embryo (F2, crown–rump length:11.1 mm) and a gestational sac with a dead embryo (F1, crown–rump length:3 mm) implanted into the lower segment Cesarean section scar in Case 9 at 7 + 4 weeks

When the following sonographic criteria were all met, the diagnosis of CSP was made [3, 12, 13, 15–17]: (1) the uterine cavity was empty; (2) empty cervical canal without a gestational sac; (3) a gestational sac showing the ‘double ring sign’ with or without cardiac activity located anteriorly at the level of the internal os; (4) a mixed mass or a clear gestational sac embedded at the lower uterine segment, within the myometrium and the fibrous tissue of the previous Cesarean section scar, and it was separated from the endometrial cavity or fallopian tube; (5) a visible myometrial defect between the bladder and the sac and a discontinuity in the anterior wall of the uterus being demonstrated on a sagittal view of the uterus running through the amniotic sac; (6) the presence of increased peritrophoblastic or periplacentalvascularity around the location of previous Cesarean section scar on color Doppler examination; (7) negative ‘sliding organs sign’, which refers to the position of gestational sac could not be changed by gentle pressure applied by the transvaginal probe (Fig. 1). When an intrauterine pregnancy (IUP) coexisted with another pregnancy which satisfied all except (1) criteria mentioned above, the diagnosis of HCSP was made. The myometrial thickness between CSP and the bladder was measured in the meantime (Fig. 2).

The management plans were formulated according to the size, gestational age (GA), peritrophoblastic vascularity and viability of CSP. Clinical symptoms, myometrial thickness, as well as patients’ wishes, were also taken into consideration. The expectant treatment might be considered if the women with minimal clinical symptoms and there is ultrasound demonstrated non-viable pregnancies. The medical treatment was appropriate when the patient was pain free, haemodynamically stable with unruptured CSP and those with signs of abnormal placentation involving the myometrium [15, 18]. The surgical treatment was offered to women who had failed non-surgical treatment or experienced heavy bleeding. All patients were informed of the severity of CSP and gave informed consent before treatment. Conservative treatment was the primary choice since all patients had a desire for continued fertility. This study was reviewed and approved by the institutional review board of the Reproductive and Genetic hospital of Citic- Xiangya.

In this study, surgical evacuation of CSP included hysteroscopy, TVS guidance dilatation and curettage (D&C). Laparotomy with wedge excision of CSP was employed as a remedy in one case due to a large amount of vaginal bleeding after methotrexate (MTX) treatment. Systemic MTX (50 mg/cm^2^) and uterine artery embolization (UAE) were used as a pretreatment in some cases. High intensity focused ultrasound (HIFU) combined with suction curettage were used on one patient. And HIFU treatment was carried out using the Haifu JC-200 focused ultrasound tumor therapeutic system (Chongqing Haifu Tech Co., Ltd., Chongqing, China). In the HCSP cases, if the ectopic CSP was an empty gestational sac, or with no viable embryo, the patients would be treated expectantly; if the CSP had detectable cardiac activity, selective embryo reduction in situ with sonographic guidance would be chosen, and potassium chloride (0.1–0.2 nmol) intracardiac injection was used. β-hCG testing and TVS scan were performed every 3–5 days in CSP patients during hospitalization and weekly thereafter, until β-hCG < 5 mIU/mL and no peritrophoblastic blood flow could be detected. In heterotopic cases, a TVS scan was performed every 3–5 days to confirm the treatment effects of CSP and observe the IUP during hospitalization and weekly thereafter. If expectant management or selective embryo reduction was applied, the retained mass of CSP would be assessed.

## Results

Over a period of four years, 12 secondary infertility patients were found to have pregnancies implanted into a previous lower segment Cesarean section scar in our hospital, and 6 (50 %) of which were HCSP cases. The incidence of CSP was 1:1688 (12/20256) and HCSP was 1:3376 (6/20256) in this study. The maternal age (MA) of the 12 patients was 33.75 (range, 27–41) years. All the 12 patients had one previous Cesarean section and the time interval between last Cesarean section and the current CSP ranged from 3–17 years. An incision lacuna had been detected in 4 patients under TVS before IVF. The infertility factors included tubal factors, ovulatory disorders, an abnormal pregnancy history, endometriosis and male factors. IVF procedures had been conducted in all the 12 patients, IVF in 7 patients, ICSI in 4 patients, IVF plus ICSI in 1 patient. Two to three high quality fresh or frozen embryos had been transferred into each patient and a total of 25 embryos had been transferred, 9 of gradeI, 16 of gradeII. The mean Day 14 β-hCG of CSP (except HCSP) after transfer was 308.15 (range, 165.3–483.8) mIU/mL, and 1012.45 (range, 558.9–1656) mIU/mL of HCSP. Eight patients complained of vaginal bleeding, and 4 of the patients suffered abdominal pain in addition to this. Two patients complained of only abdominal pain, but there were no symptoms in the remaining 2 patients (Table 1).

**Table 1:** Characteristics of 12 patients with first trimester Cesarean scar pregnancies

Case no.	Age (years)	BMI (kg/m2)	Smoker	Gravidity & parity	Previous LSCS(n)	Time interval from last C/S (years)	Infertility factor	Detectable incision lacuna by TVS before IVF	Transfer cycle	IVF techniques	Transfered embryo no.	Transfered embryo grade	Endometrial thickness on transfer day(mm)	D14β- hCG after transfer(IU/L)	Vaginal bleeding	Abdominal pain
1	30	21.5	No	G2,P1	1	3	tubal + ovulatory disorder	Yes	1	ICSI, D3transfer	2	II,II	10.2	281	-	-
2	33	22.7	No	G2,P1	1	9	tubal + male	No	1	IVF, D3transfer	2	I,II	20.2	367.3	+	-
3	39	20.02	No	G4,P2	1	17	tubal	Yes	2	IVF, D3 + D5transfer	2	I,II	11.1	235.5	+	-
4	41	24.97	No	G2,P1	1	9	tubal + male	No	2	ICSI,FET	2	II,II	10.3	483.8	+	+
5	27	25.47	No	G1,P1	1	8	tubal + endometriosis	Yes	1	IVF, D3transfer	2	II,II	13.8	316	+	+
6	33	21.09	No	G1,P1	1	5	male	No	2	ICSI, D3transfer	2	II,II	16.3	165.3	+	-
7	34	20.2	No	G2,P1	1	10	tubal	No	1	IVF + ICSI, D3transfer	3	I(1)^a^, II(2)^a^	14.7	935.2	+	+
8	32	18.21	No	G4,P1	1	5	tubal	No	1	IVF, D3transfer	2	I,I	14.2	980.4	-	+
9	38	20.17	No	G4,P1	1	10	tubal + male	No	1	ICSI, D3transfer	2	II,II	12.1	843.2	-	+
10	28	23.52	No	G2,P1	1	10	tubal + ovulatory disorder + abnormal pregnancy history	Yes	1	IVF, D3transfer	2	I,I	13.2	558.9	-	-
11	36	21.83	No	G4,P1	1	8	tubal	Yes	1	IVF, D3transfer	2	I,II	13.6	1101	+	-
12	34	23.3	No	G4,P1	1	8	tubal	No	1	IVF,FET	2	I,II	11.2	1656	+	+

*BMI* body mass index, *C/S* cesarean section, *IVF* in-vitro fertilization, *LSCS* lower segment Cesarean sections, *D* day, *ICSI* intracytoplasmic sperm injection, *FET* frozen embryo transfer, *β-hCG* beta-human chorionic gonadotropin

^a^Refers to the number of transferred embryos with different grade

All of the 12 cases with CSP were diagnosed in the first trimester by TVS. The GA ranged from 5 + 3 to 7 + 4 weeks in all CSP cases, from 5 + 6 to 7 + 4 weeks in HCSP at diagnosis. On ultrasound examination, 6 gestational sacs had fetal pole (range, 2.0–5.4 mm) and cardiac activity showed in 4 of these cases. The myometrial thickness between CSP and bladder ranged from 2.7 to 7.4 mm. Five (42 %) patients were initially treated surgically, two medically (16 %), and five expectantly (42 %).

In the surgical group, MTX, UAE and HIFU ablation were used to prevent massive hemorrhage during the surgical procedures in Case 3, Case 5 and Case 6. Case 6 received one session of HIFU ablation under conscious sedation, and suction curettage under hysteroscopic guidance was performed 2 days later. No product of conception was retained in any of the patients after surgical management. In the medical group, the success rate was 50 %. Case 11 who had a HCSP was successfully treated with TVS guidance local KCL injection to reduce the live embryo of CSP. However, the IUP terminated at 14 weeks and TVS guidance D&C was then implemented in Case 11. Case 1, who was initially treated with TVS guidance local MTX plus systemic MTX suffered heavy vaginal bleeding (>1000 mL) as well as abdominal pain, so was classified as failed, and an emergency laparotomy with a wedge excision of CSP was performed at 6 weeks to evacuate the CSP.

In the expectant group, the CSP was an empty sac in Case 8 and Case 12, only with a yolk sac in Case 7 and Case 10, a fetal pole measured 3 mm without cardiac activity in Case 9 upon diagnosis. Embryo development cessation of CSP was diagnosed by subsequent TVS detection and expectant management was chosen in these 5 patients with HCSP. Two babies were born in this group. Case 7 experienced a large amount of vaginal bleeding due to complete placenta previa, and a healthy boy weighing 2600 g was delivered by an emergency Caesarean section at 35 weeks’ gestation. Surgical exploration of the scar found an amorphous mass (21 × 14 mm) which was removed intraoperatively, and deciduous tissues were found during the pathological examination. The development of IUP was uneventful in Case 10 and a healthy girl, weighing 2900 g, was delivered by Caesarean section at 36 weeks’ gestation due to the premature rupture of membranes. And the scar mass disappeared at 22 weeks’ gestation in Case 10.

TVS guidance D&C was performed in Case 8 because of IUP termination at 13 weeks’ gestation. Case 9 experienced a second trimester abortion (6 months’ gestation). The IUP of Case 12 remained viable and progressed uneventfully until the time of writing (18 weeks’ gestation) and there was still a detectable ectopic mass (32 × 27 mm) under TVS. Although full-term birth was not achieved in Case 8, Case 9 and Case 12, the management of these three cases were classified as successful due to the particularity of HCSP. Therefore, the success rate was 100 % (5/5) in the expectant group.

In the patients who were successfully treated, the β-hCG resolution time was between 26 and 52 days with the exception of HCSP. And the disappearance time of peritrophoblastic flow after treatment ranged from 30 to 118 days (mean 61 days), from 44 to 118 days of HCSP patients (mean 76 days). And the uterus was preserved in all the 12 patients (Table 2).

**Table 2:** Clinical diagnosis and treatment of 12 patients with first trimester Cesarean scar pregnancies

Case no.	Gestational age at diagnosis (weeks)	Type of pregnancy	Gestational sac of CSP(mm)	CRL(mm)	Viability	Myometrtrial thickness(mm)	Type of treatment	Success	Time after treatment for β-hCG to reach normal level (days)	Time after treatment for subtrophobla-stic flow to disappear (days)	Follow-up information
1	6 + 3	CSP	22 × 12	3.6	Viable	2.9	TVS guidance local MTX + Systemic MTX	No	**-**	**-**	Laparotomy with wedge excision of CSP at 6 W+ due to massive vaginal bleeding and abdominal pain after MTX treatment
2	5 + 3	CSP	6 × 5	no	Non-viable	4.7	Hysteroscopy	Yes	38	42	Cured, no attempt at another pregnancy
3	6 + 2	CSP	18 × 14	2	Viable	4.5	Systemic MTX + TVS guidance D&C	Yes	52	49	Cured, waiting for another IVF treatment
4	5 + 6	CSP	16 × 10	no	Non-viable	3.7	Hysteroscopy	Yes	32	41	Cured, no attempt at another pregnancy
5	7 + 0	CSP	27 × 17	5.4	Viable	3.3	UAE + Hysteroscopy	Yes	44	57	Cured, no attempt at another pregnancy
6	5 + 4	CSP	4.8 × 3.4	no	Non-viable	4.9	HIFU + Hysteroscopic guidance suction curettage	Yes	26	30	Cured, no attempt at another pregnancy
7	6 + 5	Heterotopic	16 × 7	no	Non-viable	5.9	Expectant	Yes	**-**	77	Cesarean section due to massive hemorrhage caused by complete placenta previa at 35 W+,ectopic mass (21 × 14 mm) was removed at the same time, and a healthy boy weighing 2.6 kg was born
8	5 + 6	Heterotopic	19 × 6	no	Non-viable	2.7	Expectant	Yes	**-**	52	TVS guidance D&C due to IUP termination at 13 W+
9	7 + 4	Heterotopic	20 × 12	3	Non-viable	5.2	Expectant	Yes	**-**	96	Second trimester abortion (6 months’ gestation) of IUP
10	6 + 3	Heterotopic	4 × 3	no	Non-viable	7.4	Expectant	Yes	**-**	69	Cesarean section due to premature rupture of membranes at 36 W+, a healthy girl weighing 2.9 kg was born; Scar mass disappeared at 22 W+
11	6 + 3	Heterotopic	39 × 8	3.4	Viable	4.2	TVS guidance local KCL	Yes	-	44	TVS guidance D&C due to IUP termination at 14 W+
12	7 + 1	Heterotopic	27 × 10	no	Non-viable	4.1	Expectant	Yes	-	118	IUP was 18 W+ gestation at the time of writing; Retained ectopic mass (32 × 27 mm)

*CSP* Cesarean scar pregnancy, *CRL* crown—rump length, *TVS* transvaginal ultrasound guidance, *MTX* methotrexate, *D&C* dilatation and curettage, *UAE* uterine artery embolization, *HIFU* high intensity focused ultrasound, *KCL* potassium chloride, *IUP* intrauterine pregnancy

## Discussions

The incidence of CSP was 1:1688 in this study, which seemed higher than previous reports [3, 7]. Moreover, the ratio of HCSP/CSP increased to 50 %, which was much higher than spontaneous condition, suggesting IVF could be a contributor to the occurrence of CSP and could greatly increase the likelihood of HCSP. A question may be raised why CSP occurred in IVF-ET, since the embryos were transferred directly into the uterine cavity. We could also ask whether there is an association between CSP and IVF-ET technology. The occurrence of CSP might be explained by the ectopic tract which has been previously mentioned [3, 4]. And it was reported that frozen cycles were associated with lower rates of EP compared with fresh cycles [19]; Frozen-thawed Day 5 blastocyst transfer was associated with a lower EP rate than frozen-thawed Day 3 transfer and fresh transfer [20]; Higher EP rates in GnRH agonist triggered cycles relative to hCG triggered cycles [21]; The endometrial thickness was thinner in patients with an EP [22], and so on. Then, whether fresh and frozen cycles, IVF and ICSI, Day 3 and Day 5 transfer, and the classification of embryo quality, etc. have different effects on the occurence of CSP? Although there is still no study focusing on the relation between CSP and IVF-ET technology, we are not able to analyze it here due to the small number of patients. A larger sample is needed for further study.

Many reports have expressed the notion that CSP cases are more likely to happen after multiple Cesarean sections [3, 5, 7]. But all the 12 patients had experienced only one Cesarean section due to the Family Planning Policy in China. Accordingly, the correlation between multiple Cesarean sections and CSP cannot be discussed under such circumstances in this study.

Vaginal bleeding and mild to moderate abdominal pain were the most common symptoms in the 12 patients, and 33.3 % (4/12) patients presented both of these. But the manifestations were not specific. There were two cases without any symptoms which were diagnosed by routine Day 28 TVS examination. Therefore, the lack of symptoms does not mean CSP is necessarily avoided. Previous studies have suggested that most cases of HCSP were without any symptoms and diagnosed by routine first-trimester sonography [23, 24]. But in this study, 83.3 % (5/6) patients with HSCP were diagnosed based on clinical symptoms. And the difference means a large sample is needed for further study.

An effort to diagnose CSP early is always made in women with a history of Cesarean section. High-resolution TVS appears to be the best diagnostic tool, as it is highly efficient in the diagnosis of CSP. The size, shape and position of CSP can be detected via a two dimensional TVS scan. In addition, the blood flow signals around CSP and fetal cardiac activity can be visualized through the color doppler flow index (CDFI). When profuse vascularity shows around the location of CSP, the patients should be informed of the risk of uterine rupture [3], and corresponding treatment should be applied immediately. Moreover, CSP should be differentially diagnosed with low IUP, cervical pregnancy and miscarriage. It is relatively simple to differentiate them early in pregnancy, but as the pregnancy progresses, the distinction becomes more difficult. All the 12 patients were diagnosed in the first trimester (range, 5 + 3 to 7 + 4 weeks), the early diagnostic time was attributed to the utilization of TVS and routine TVS examination after IVF-ET. But there were 4 patients who were diagnosed later than Day 28 due to having received some clinical treatments to deal with the vaginal bleeding and/or abdominal pain, and therefore postponed their TVS scan time.

TVS plus Day 14 β-hCG are important diagnostic methods after IVF-ET. And Day 14 β-hCG is useful in predicting the pregnancy outcome. In this study, the level of Day 14 β-hCG in CSP was similar to a normal single IUP, and the level in HCSP was similar with twin IUP or HP. Therefore, Day 14 β-hCG alone has little value in determining the location of CSP. When the level of Day 14 β-hCG is high, it is necessary to exclude a coexisting EP after the diagnosis of IUP has already been made. And CSP should be carefully excluded in patients with a history of Caesarean section.

The majority of Cesarean scars are well-healed, but visible deficiency may be detected in a small number of women due to fibrosis cause poor vascularity of the lower uterine segment which in turn affects the scar postoperative healing [7]. We need to determine whether it is necessary to repair the incision lacuna. Surgical repair before the next conception was recommended by some authors [25, 26]. However, other authors hold a contrary opinion that surgical repair itself was a trauma to patients and it might not prevent the occurrence of uterine rupture in the subsequent pregnancy [3, 27]. Incision lacuna was detected in 5 patients and the Cesarean scars were well-healed in the other 7 cases in this study, suggesting CSP might also occur in well-healed Cesarean scars, therefore, the necessity of surgical repair was questionable.

The previous literature has shown that complications such as miscarriage, EP, abruptio placentae, placenta previa and placenta accreta are more likely to happen in women with a history of Cesarean section [28]. In this study, there were two babies born, however, one with complete placenta previa and the other with premature rupture of membranes. And 66.67 % (8/12) cases of CSP ended in spontaneous first-trimester miscarriages which seemed higher than the findings of a previous study [7]. The first-trimester miscarriage rate could be higher if all cases were managed expectantly. However, the mean MA was 33.75 years which might be another contributor to this high rate.

Early diagnosis makes early treatment possible which may reduce complications and improve the treatment success rate. All patients in our center have fertility requirements, so it is important to take relatively conservative approaches to preserve the uterus. However, with limited experience of CSP in the first trimester, it is difficult to decide on optimal management. There were 2 cases that were managed medically, and the success rate was 50 %. Local + system MTX treatment failed in Case 1 and expedient laparotomy had to be implemented due to extensive vaginal bleeding. And it was the only one case treated through laparotomy in this study. Stevens EE [29] had reported a case with massive haemorrhage postoperative after the treatment of a combination of local and system MTX injection. However, most previous studies regarded MTX especially local MTX injection as an effective management technique [30, 31].

The success rate of the medical group in this study was not representative due to the small sample involved. There are risks of profuse haemorrhage and uterine rupture when only treated medically [29], thus many doctors prefer combined treatment. In fact, some cases in the surgical group were combined with medical therapy in this study.

The patients of the surgical group were all successfully treated transcervically. MTX and UAE were effectively used to prevent intraoperative bleeding before the operation. Owing to the strong demands of patient, HIFU was tentatively applied in Case 6. HIFU is a relatively new technique which has been used in recent years. It can convert acoustic waves to thermal energy at the focal point where the temperature increases over 65 °C and coagulative necrosis occurs. It has been demonstrated that it can be used to treat various diseases successfully, such as breast tumors, hepatocellular carcinomas, bone malignancy, pancreatic cancers [32–35], uterine fibroids [35, 36], adenomyosis [37] and so on. Over the past few years, there have been some studies focusing on the treatment of CSP by this non-invasive technique, which consider HIFU to be an effective method [38–40]. In addition, it can apparently decrease the risk of intraoperative hemorrhage [40]. The application of HIFU in this study was successful, and no obvious complications were observed. There were relatively short time for β-hCG to reach normal level and peritrophoblastic flow to disappear after HIFU ablation. And the same method was also successful in 35 patients in the Xiaogang Zhu’s [38] study. But investigations with a larger sample size are necessary to further confirm its effectiveness in treating CSP.

The ratio of HCSP/CSP was quite high in this study. We considered the reason to be partly attributable to the application of IVF-ET, and partly attributable to coincidence. It is troublesome to deal with CSP, not to mention whilst simultaneously protecting the IUP. Expectant treatment is not encouraged due to the catastrophic risk of CSP [3, 7]. Jurkovic D. [7] has reported 2 failed cases in 3 patients who were treated expectantly. There were 5 (41.67 %) patients with HCSP managed expectantly in this study, the IUP of which went through the first trimester smoothly under close observation, and consequently, two babies were born. It suggests that expectant management under close observation may be an appropriate choice when the development of the CSP in HCSP was confirmed to have a termination. And this opinion had been advocated by some researchers [41, 42]. However, it didn’t mean that expectant management could be used in all HCSP, when fetal cardiac activity was detected, just as was the case in Case 11 in this study, the selective embryo reduction in situ may be appropriate, and it had been demonstrated in some of the previous literatures [43, 44].

## Conclusions

In summary, CSP is rare but can cause life-threatening complications. And the Caesarean section and IVF-ET may increase the ratio of HCSP/CSP. When the diagnosis and treatment of CSP are made in the first trimester, the prognosis is better and the fertility of most patients as well as the IUP of HCSP is more likely to be preserved.

## Abbreviations

CDFI: color doppler flow index
CSP: Caesarean scar pregnancy
D&C: dilatation and curettage
EP: ectopic pregnancy
GA: gestational age
HCSP: heterotopic CSP
HIFU: high intensity focused ultrasound
IUP: intrauterine pregnancy
IVF-ET: in-vitro fertilization-embryo transfer
MA: maternal age
MTX: methotrexate
TVS: transvaginal sonography
UAE: uterine artery embolization
β-hCG: beta human chorionic globulin
